# PONYTA: prioritization of phenotype-related genes from mouse KO events using PU learning on a biological network

**DOI:** 10.1093/bioinformatics/btae634

**Published:** 2024-10-21

**Authors:** Jun Hyeong Kim, Bonil Koo, Sun Kim

**Affiliations:** Interdisciplinary Program in Artificial Intelligence, Seoul National University, Seoul 08826, Republic of Korea; Interdisciplinary Program in Bioinformatics, Seoul National University, Seoul 08826, Republic of Korea; AIGENDRUG Co., Ltd., Seoul 08758, Republic of Korea; Interdisciplinary Program in Artificial Intelligence, Seoul National University, Seoul 08826, Republic of Korea; Interdisciplinary Program in Bioinformatics, Seoul National University, Seoul 08826, Republic of Korea; AIGENDRUG Co., Ltd., Seoul 08758, Republic of Korea; Department of Computer Science and Engineering, Seoul National University, Seoul 08826, Republic of Korea

## Abstract

**Motivation:**

Transcriptome data from gene knock-out (KO) experiments in mice provide crucial insights into the intricate interactions between genotype and phenotype. Differentially expressed gene (DEG) analysis and network propagation (NP) are well-established methods for analysing transcriptome data. To determine genes related to phenotype changes from a KO experiment, we need to choose a cutoff value for the corresponding criterion based on the specific method. Using a rigorous cutoff value for DEG analysis and NP is likely to select mostly positive genes related to the phenotype, but many will be rejected as *false negatives*. On the other hand, using a loose cutoff value for either method is prone to include a number of genes that are not phenotype-related, which are *false positives*. Thus, the research problem at hand is how to deal with the trade-off between false negatives and false positives.

**Results:**

We propose a novel framework called PONYTA for gene prioritization *via* positive-unlabeled (PU) learning on biological networks. Beginning with the selection of true phenotype-related genes using a rigorous cutoff value for DEG analysis and NP, we address the issue of handling false negatives by rescuing them through PU learning. Evaluations on transcriptome data from multiple studies show that our approach has superior gene prioritization ability compared to benchmark models. Therefore, PONYTA effectively prioritizes genes related to phenotypes derived from gene KO events and guides *in vitro* and *in vivo* gene KO experiments for increased efficiency.

**Availability and implementation:**

The source code of PONYTA is available at https://github.com/Jun-Hyeong-Kim/PONYTA.

## 1 Introduction

Unveiling the association between genes and phenotypes is crucial, prompting gene knock-out (KO) experiments in mice. Genes within a gene interaction network engage in complex interactions, comprising various biological pathways. A genotypic change resulting from a gene KO event propagates through cascade effects on the gene interaction network, leading to phenotype alteration. Therefore, comparative analysis of transcriptome data from wild-type (WT) and gene KO conditions enables understanding of genes related to phenotype changes induced by gene KO.

### 1.1 Challenge

Differentially expressed gene (DEG) analysis elucidates genes correlated with a particular phenotype under gene KO conditions and ranks genes associated with a phenotype. By analysing gene expression profiles obtained from high-throughput technology under two different conditions, it ranks genes based on different expression values verified through statistical tests (Anders and Huber 2010). DEG analysis evaluates the relationship between genes and phenotypes based solely on their distinct expression values without considering interactions between them. This makes it challenging to detect genes with subtle changes in expression levels, such as transcription factors, which regulate the expression of other genes rather than their own (Hur *et al.* 2015). Furthermore, many researchers emphasize the intricate relationship between genotype and phenotype, insisting that attributing phenotype differences solely to the alteration of a single gene is inherently ambiguous (Wang *et al.* 2008). Additionally, DEG analysis is prone to generating false positive results, influenced by variables such as the size of replicate samples or experimental conditions (Schurch *et al.* 2016).

Network propagation (NP), assuming neighboring genes share responsibility for similar phenotypes, considers genetic associations within a gene interaction network by propagating information across their cross-linking edges (Cowen *et al.* 2017). Utilizing provided seed genes to initiate the information propagation process, NP ranks functionally related genes by amplifying biological information on the network employing a random walk with restart algorithm. For example, in identifying key regulators of adipocyte senescence in obesity, NP was successfully utilized to reveal SREBF1 as a prominent transcription factor (Lee *et al.* 2022). However, NP suffers from yielding false positive genes, due to its ‘exclusive’ dependency on network topology or noise within the gene interaction network without considering genes expression values.

Both DEG analysis and NP, as gene prioritization methods, require setting a cutoff value for selecting genes related to the phenotype from the gene rank. It is crucial to select a reasonable cutoff value in order to obtain genes truly related to the phenotype. Using a flexible cutoff value leads to a greater possibility of incorporating *false positive genes*. Conversely, using a strict cutoff value results in a decreased number of *false positive genes* but an increased number of *false negative genes* due to the trade-off. Thus, it is essential to properly address the trade-off problem when selecting cutoff values to ensure that the genes chosen are genuinely related to the phenotype. Furthermore, each gene prioritization method recommends genes related to the phenotype according to distinct criteria. This makes it ambiguous identifying negative genes, which are not considered potential candidates for positive genes, within the entire gene interaction network. Therefore, leveraging a deep learning method for gene prioritization that can learn without requiring fully labeled genes would be efficient in practice.

### 1.2 Approach

**Figure 1.**
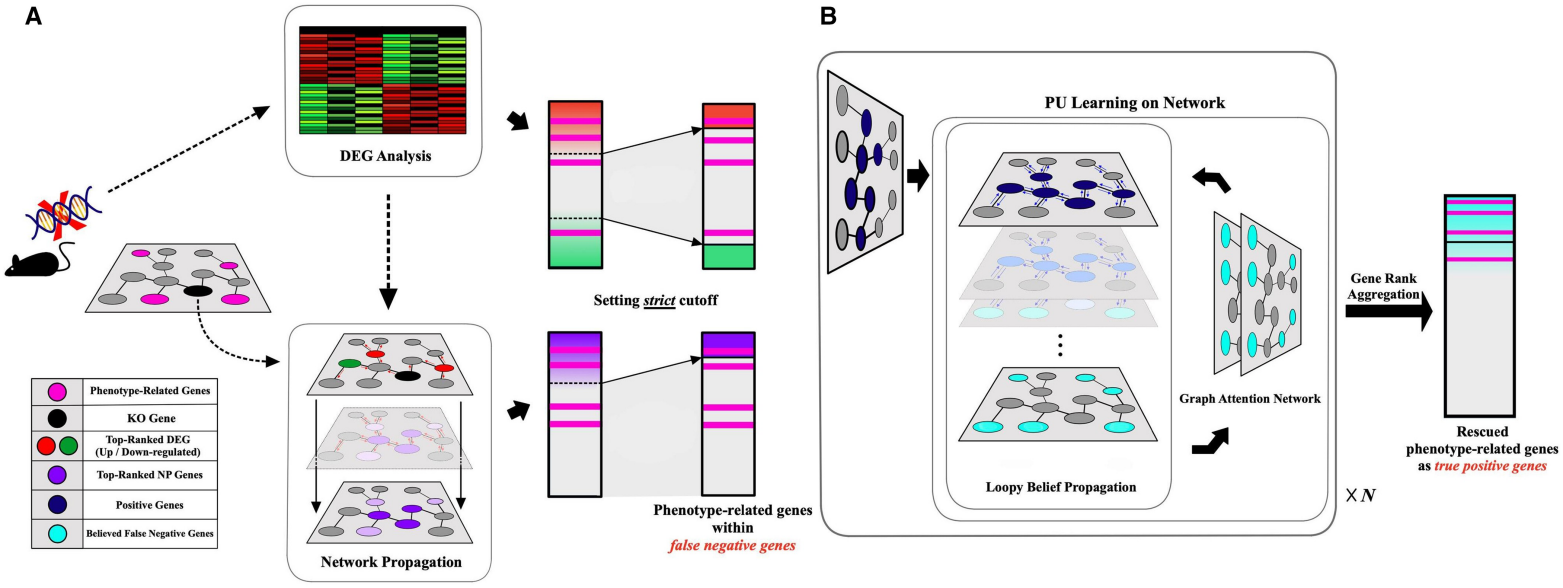
Overview of ranking algorithm for phenotype-related genes using PU learning on a biological network. (A) DEG analysis and NP are employed to obtain top-ranked positive genes related to the KO gene. For NP, both the KO gene and the top-ranked genes from DEG analysis are used as seed genes. (B) PU learning applied is applied to a biological network to rank genes, using the top-ranked genes from data the initial data preprocessing as input positive genes. The process involves iterations between Loopy Belief Propagation and updates of graph attention network, refining the probability of unlabeled genes being positive genes. Unlabeled genes with large Belief values, indicating their probability of being part of the positive gene set, are identified as *Believed false negative genes*. PU learning outputs a ranked list of genes based on their probability of being positive. Through gene rank aggregation, a single gene list is created by combining rank information from each iteration of PU learning, resulting in a comprehensive and refined list of phenotype-related genes. PU learning, positive-unlabeled learning; DEG, differentially expressed gene; KO, knock-out; NP, network propagation.

We propose a novel approach, PONYTA (POsitive-uNlabeled learning on biological network for phenotYpe-relaTed genes prioritizAtion), as a prioritization algorithm for genes functionally related to phenotypes within specific gene KO conditions using positive-unlabeled (PU) learning on biological networks (Fig. 1). Unlike most traditional classification learning methods, PU learning trains with only a subset of labeled positive examples without needing labeled negative samples and is evaluated based on its ability to predict remaining portion of positive examples (Bekker and Davis 2020). Due to these characteristics, several studies have adopted PU learning as a recommendation system, prioritizing or recommending items that exhibit properties of positive samples among unlabeled ones (Liang *et al.* 2016, Saito *et al.* 2020). In this study, considering a scenario where only positive genes related to the phenotype are available, we leveraged PU learning on the biological network using only reliable positive genes to prioritize genes from the unlabeled set that are likely associated with the phenotype.

Using top-ranked genes from DEG analysis and NP as positive genes allows PU learning to incorporate both expression patterns and cascade effects among them and minimizes the risk of erroneously considering false positive genes as positive genes. Consequently, we rescue *phenotype-related genes*, revealing false negative genes among unlabeled genes considering both individual and cooperative properties among reliable affected genes under KO conditions. We evaluated the prioritization ability of our framework on phenotype-related genes obtained from gene KO studies using either single-cell RNA sequencing or bulk RNA sequencing and outperformed benchmark models. We conducted studies to demonstrate the robustness by varying the number of positive genes and gene rank aggregation methods on output lists of genes from each iteration. We also selected different portions as positive genes other than the top portion, showing that using reliable genes as positive genes enables superior gene prioritization ability of our approach. Through functional analysis, we found highly-ranked genes are functionally associated with the phenotype results from gene KO conditions. Thus, PONYTA contributes to the prioritization of genes functionally related to phenotypes within gene KO conditions, providing guidance for subsequent *in vivo* and *in vitro* biological experiments for further validation of genes functions.

## 2 Materials and methods

### 2.1 Data preprocessing

We explored gene KO studies within mice on the Gene Expression Omnibus (GEO) and European Nucleotide Archive (ENA) databases,  and processed gene expression data for both WT and KO conditions of specific genes. Depending on the study type, different processes were used to obtain the gene expression data (Supplementary Information S1). To acquire positive genes for PU learning, we first performed DEG analysis between the two preprocessed gene expression profiles from WT and KO conditions. Depending on the study type, we used Seurat (Hao *et al.* 2024) based on the Wilcoxon rank sum test for single-cell RNA sequencing and DESeq2 (Love *et al.* 2014) for bulk RNA sequencing. We filtered genes with an absolute log2FoldChange (log2FC) value greater than 1 and an adjusted *P*-value below .05, subsequently reordered them in ascending order of adjusted *P*-value. Among the selected DEGs, we set only the top 50 DEGs as positive genes in order to minimize the risk of false positives. To properly account for cascade effects among genes, which are difficult to manage solely based on gene expression values, we also conducted NP on the STRING (Szklarczyk *et al.* 2023) network to obtain additional positive genes. For NP, we placed the KO gene along with the top 50 DEGs as seed nodes to initiate the propagation process. From the NP results, where all genes within the network were ranked based on their NP scores, we additionally set the top 50 genes as positive genes.

### 2.2 PU learning on gene interaction network

To apply PU learning on the gene network, we employed GRAB (Yoo *et al.* 2021), the method specifically designed for graph data S=(G,E), where G is the set of nodes representing genes and E is the set of edges between genes. Interpreting the graph as a pairwise Markov network allows us to model the probabilistic connections between genes. This approach leverages the Markov network properties to consider structure-based relationships, enabling the effective propagation of information between labeled and unlabeled genes.

The method consists of two main algorithms for PU learning on a network, the *marginalization step* and the *update step*.

#### 2.2.1 Marginalization step

For each unlabeled gene, latent variables are introduced to indicate the probability of the gene being positive. The joint probability of these latent variables is marginalized utilizing both the gene information itself and the edge information from neighboring genes. Each element in this process corresponds to node potential and edge potential. Node potential, set to the updated approximated prior value ${\hat{\pi }}_{p}$, implies the prior probability of a gene being positive, independent of other genes. Initially, this is set to 0, assuming all unlabeled genes are negative due to the absence of a pre-defined value. Edge potential, ranging from 0 to 1, implies the degree of homophily between two different genes.

Given the large size of the gene interaction network, directly deriving marginalization involving expectation calculations is computationally extensive, making the learning process impractical. Therefore, instead of explicit derivation, GRAB employs Loopy Belief Propagation (LBP) to obtain the approximated marginal distribution. Throughout the propagation process, *messages* are updated between every pair of genes, representing the probability of a gene being positive or negative genes as estimated by its neighboring genes. Once the message values have converged, the *belief* values $b_{j}(u)$, which are the approximated marginal probability values for genes, can be calculated, and a belief matrix *B* is constructed for all genes within the network.

#### 2.2.2 Update step

The belief matrix is then used as a soft-label for unlabeled genes during the *update step* to optimize the parameter θ for a binary classifier with the objective function $\tilde{L}$:

(1) $$\tilde{L}(\theta ;X,y,B,R,U)=\frac{1}{\left|R\right|}\sum_{i\in R}l({\bar{y}}_{i},{\hat{y}}_{i})+\frac{1}{\left|U\right|}\sum_{j\in U}l(b_{j},{\hat{y}}_{j})$$

Here, ${\bar{y}}_{i}$ is the one-hot label vector for gene $y_{i}$ within the positive genes set R, and ${\hat{y}}_{j}$ is the probability vectors for gene $y_{j}$, obtained through graph attention network (Veličković *et al.* 2017), within the unlabeled genes set U. The loss function l can vary, but we specifically employed the negative log likelihood function.

Upon updating the parameter θ, the approximated prior value ${\hat{\pi }}_{p}$, which represents the predicted ratio of positive genes within the unlabeled genes, is also updated to ${\hat{\pi }}_{p}^{\prime }$ based on the following formula:

(2) $${\hat{\pi }}_{p^{\prime }}=\frac{1}{\left|U\right|}\sum_{j\in U}I[{\hat{y}}_{j}(+1;\theta^{\prime })>0.5]$$

This updated prior value is then employed in the marginalization step for subsequent iterations. The trained learner produces a ranked list of genes, sorted in decreasing order of their probability of being positive genes. The detailed procedure of PU learning on networks can be found in Supplementary Information S2.

### 2.3 Gene rank aggregation

Studies on ranking genes related to biological events have highlighted the importance of aggregating multiple gene ranks from different experiments to gain a deeper understanding of the underlying mechanisms and to reduce biases stemming from diverse experimental conditions (Wang *et al.* 2015). For aggregating gene rank lists, each generated through PU learning using different fold sets of input positive genes, we employ weighted DIBRA, an iterative distance-based aggregation method, with implementation provided by FLAGR (Akritidis *et al.* 2022, 2023). Measuring the distances between aggregated gene list and individual gene rank lists obtained from PU learning on each fold, weighted DIBRA assigns weights to the individual gene rank lists based on the distances. Once the weight values are determined, the final aggregation process is performed across the gene ranked lists, with genes in the lower portion pruned. Since the key objective of PONYTA is to rank phenotype-related genes in highly-ranked portion, the pruning process aligns with our goals. The detailed procedure of weighted DIBRA is provided in the Supplementary Information (Supplementary Information S3).

## 3 Experiment

### 3.1 Evaluation metric

We evaluated whether the prioritizing method is capable of ranking phenotype-related genes as highly-ranked genes and compared its performance with other benchmark models. We adopted the partial AUC metric which measures the area under the ROCn curve. Unlike general ROC curves, the ROCn curve plots the rate of positive genes ranked higher than the nth highest ranked negative gene (Scott and Barton 2007, Dimitrakopoulos *et al.* 2018). The formula to derive partial AUC is as follows:

(3) $$AUC_{n}=\frac{1}{nR}\sum_{i=1}^{n}R_{i}$$

where n is the number of highly-ranked negative genes to cover, R is the total number of phenotype-related genes acquired from the study, and $R_{i}$ is the number of phenotype-related gene that ranked higher than ith highest scored negative gene. As noted by Scott and Barton (2007), the partial AUC value is low since phenotype-related genes make up a small portion of the total genes in the network.

### 3.2 Dataset

To assess the effectiveness of our method in ranking phenotype-related genes, we compiled RNA-Seq datasets from various studies involving gene KO experiments (Table 1). Analysing the papers that produced datasets we used, each focused on genes regulated by or associated with specific gene KO event, we curated phenotype-related genes as ground truth genes (Supplementary Tables S1–S5). Since the whole set of genes associated with KO events is inherently unknown, these curated genes were employed to evaluate whether our method is capable of ranking such genes prominently. We referred to studies conducted using either single-cell RNA sequencing or bulk RNA sequencing to examine the generalization capabilities of PONYTA across different types of data. Phenotypes related to each gene KO study are detailed in the Supplementary Section (Supplementary Table S6).

**Table 1.** Datasets used for evaluation.

KO gene	RNA-seq type	Cell type	Accession number
Nkx2-1	scRNA-seq	Alveolar type 1 cell	GSE129584 (Little *et al.* 2019)
Nr5a1	scRNA-seq	Sertoli cell	GSE219271 (Souali-Crespo *et al.* 2023)
Tcf4	scRNA-seq	Cortical neuron	GSE147247 (Wittmann *et al.* 2021)
Pax6	bulk RNA-seq	–	PRJEB20579 (Mitchell *et al.* 2017)
Sarm1	bulk RNA-seq	–	GSE182091 (Doran *et al.* 2021)

### 3.3 Benchmark models

We assessed the ability to rank genes using several other methods for comparison with our approach. Node2vec (Grover and Leskovec 2016) generates low-dimensional vectors representing each gene within the gene network. This is achieved by minimizing the conditional log-probability of observed neighbors of genes sampled through a random walk (Zhang *et al.* 2021). Randomwalk with restart (RWR) (Köhler *et al.* 2008) measures the probability of a node being the terminal node of a path generated by a random walk process that initiates from seed nodes. DIAMOnD (Ghiassian *et al.* 2015) is a systematic algorithm that evaluates the extent of connections a node possesses with seed nodes. The assessment is carried out through the utilization of hypergeometric distribution in a step-wise manner. GenePanda (Yin *et al.* 2017) is a network analysis-based method for prioritizing disease genes. It employs the concept of adjusted network distance by considering the average distance with other genes when measuring direct distance between genes.

We utilized the top-ranked genes obtained from DEG analysis and NP, comprising 50 genes from each method, as positive genes. For the evaluation on the gene interaction network, we leveraged the STRING v12.0 (Szklarczyk *et al.* 2023) as protein–protein interaction network. To ensure interaction reliability, we selected edges with a confidence score greater than 0.700, resulting in a network of 15 080 genes with 201 141 edges. To ensure the robustness of the ranked gene lists produced by each algorithm, we first obtained gene rank lists using different sets of labeled positive genes by performing 10 times of five-fold cross-validation. The final aggregated list of ranked genes from each method was obtained *via* the weighted DIBRA method aggregation process.

## 4 Results

### 4.1 Phenotype-related genes ranking evaluation

To assess the capability of each method in ranking phenotype-related genes within the highly-ranked genes, we calculated partial AUC values. Specifically, we set the number of negative genes to cover as 300, denoted as n for $AUC_{n}$. We also evaluated the partial AUC value at $AUC_{200}$ to demonstrate the consistency in the prioritization ability. By setting a small value for n relative to the total number of genes in the gene network, we were able to emphasize the proportion of phenotype-related genes among the highly-ranked portion, which are targeted for further validation through biological experiments in practical cases. Prior calculating partial AUC values, we excluded positive genes used as input from the aggregated gene rank list to align with our objective of prioritizing genes not yet known to be associated with the KO gene.

Partial AUC values were evaluated in order to demonstrate each method’s abilities in phenotype-related genes prioritization within the highly-ranked portion (Table 2). PONYTA showed superior performance compared to other benchmark methods. RWR secured the second-highest performance following our approach within number of datasets. However, *Nr5a1* and *Sarm1* as KO genes, each case involved in different type of RNA studies, RWR failed to achieve competitive scores, while our approach successfully captured phenotype-related genes regardless of their study type. Additionally, PONYTA consistently shows high partial AUC scores for $AUC_{200}$ as well as $AUC_{300}$, indicating that the performance of PONYTA is not significantly affected by the cutoff values. We further demonstrated the qualitative and quantitative superiority of PONYTA over other baseline models using several additional metrics on the prioritized gene list (Supplementary Information S4, Supplementary Tables S7–S10). Detailed rank information of phenotype-related genes can be found in the Supplementary Section (Supplementary Tables S11–S15).

**Table 2.** Performance comparison based on partial AUC values for each method across different datasets.

Method	Nkx2-1 KO		Nr5a1 KO		Tcf4 KO		Pax6 KO		Sarm1 KO	
	$AUC_{200}$	$AUC_{300}$	$AUC_{200}$	$AUC_{300}$	$AUC_{200}$	$AUC_{300}$	$AUC_{200}$	$AUC_{300}$	$AUC_{200}$	$AUC_{300}$
Node2vec	0.0298	0.0314	0.0428	0.0553	0.0563	0.0726	0.0394	0.0548	0.0715	0.1331
DIAMOnD	0	0	0.0703	0.0926	0.0471	0.0489	0.0103	0.0281	0.0096	0.0663
RWR	0.0298	0.0494	0.0298	0.0494	0.0977	0.1403	0.1289	0.2095	0.0103	0.0281
GenePanda	0.0102	0.0299	0.0069	0.0185	0	0	0.0131	0.0504	0	0
PONYTA	**0.0769**	**0.1179**	**0.1245**	**0.1853**	**0.1961**	**0.2496**	**0.2988**	**0.3658**	**0.1312**	**0.1900**

The highest partial AUC value for each case is indicated in bold, and the second-highest partial AUC value is underlined.

### 4.2 Robustness of phenotype-related gene prioritization

To evaluate the robustness of our approach in phenotype-related genes prioritization, we measured partial AUC values while introducing slight variance in the number and ratio of input positive genes obtained from DEG analysis and NP. We considered all possible combinations between 50 and 100 genes from each method other than the pre-defined top-ranked genes ratio of 50 DEGs and 50 NP genes (Table 3). PONYTA mostly showed superior performance to benchmark models even when the number of top portion positive genes used as input was altered, while benchmark models failed to maintain steady performance, with their partial AUC values fluctuating significantly in most cases. The results also indicate that setting cutoff as arbitrary but *strict* values can ensure PONYTA to rescues false negatives within unlabeled genes, thereby addressing trade-off problem of setting cutoff values properly.

**Table 3.** Performance comparison based on partial AUC values for each method across different datasets with altered numbers of positive genes.

	Nkx2-1		Nr5a1		Tcf4		Pax6		Sarm1	
Method	$AUC_{200}$	$AUC_{300}$	$AUC_{200}$	$AUC_{300}$	$AUC_{200}$	$AUC_{300}$	$AUC_{200}$	$AUC_{300}$	$AUC_{200}$	$AUC_{300}$
# of DEGs = 50 & # of NP genes = 100										
Node2vec	0.0275	0.0421	0.0286	0.0376	0.0194	0.0315	0	0	0	0.0528
DIAMOnD	0.0177	0.0237	0.0611	0.0917	0.0486	0.0509	0.0281	0.0600	0	0.0556
RWR	0.0989	0.1352	0.0804	0.1274	**0.1828**	**0.2330**	0.0969	0.1305	0.0417	0.0794
GenePanda	0.0748	0.1068	0.0828	0.1125	0.0658	0.1020	0.0265	0.0592	0.0608	0.0683
PONYTA	**0.1180**	**0.1729**	**0.0978**	**0.1717**	0.1281	0.1976	**0.2196**	**0.2746**	**0.1663**	**0.2192**
# of DEGs = 100 & # of NP genes = 50										
Node2vec	0	0	0.0968	0.1317	0.0362	0.0400	0	0	0.0668	0.1443
DIAMOnD	0.0282	0.0307	0.0324	0.0510	0	0	0	0.0017	0.0396	0.0693
RWR	0.0493	0.0595	0.0760	0.1097	**0.0805**	0.1238	0.1379	0.1753	0.0643	0.0983
GenePanda	0.0054	0.0331	0.0224	0.0504	0.0305	0.0362	0	0.0014	0.0525	0.0826
PONYTA	**0.0862**	**0.1435**	**0.1116**	**0.1459**	0.0790	**0.1495**	**0.2842**	**0.3006**	**0.1050**	**0.1685**
# of DEGs = 100 & # of NP genes = 100										
Node2vec	0	0.0015	0.0611	0.0802	0	0	0	0	0.0362	0.0797
DIAMOnD	0.0246	0.0283	0.0184	0.0346	0	0	0	0.0178	0	0.0222
RWR	0.0354	0.0557	0.0581	0.1050	0.0711	0.1011	0.0950	0.1189	0.1083	0.1581
GenePanda	0.0070	0.0215	0.0472	0.0525	0	0.0039	0	0.0092	0	0.0086
PONYTA	**0.0898**	**0.1370**	**0.0966**	**0.1165**	**0.1268**	**0.2158**	**0.2271**	**0.3183**	**0.1638**	**0.2144**

The highest partial AUC value for each case is indicated in bold, and the second-highest partial AUC value is underlined.

Given that numerous gene rank aggregation methods are used in practice, and each method can affect the order of genes within the aggregated list, we further demonstrated the robustness of our performance with various gene rank aggregation methods. For one of the gene sets with different numbers of positive genes obtained from DEG analysis and NP, we applied alternative gene rank aggregation methods: median rank aggregation (Fagin *et al.* 2003), linear combination with Borda normalization (Renda and Straccia 2003), and the majoritarian method (Farah and Vanderpooten 2007). We then measured partial AUC values for each aggregated output list (Supplementary Tables S16–S18). Although there was a slight difference in partial AUC values for certain datasets, *Nr5a1* and *Sarm1*, PONYTA consistently achieved superior performance in the gene prioritization task.

### 4.3 Evaluation of phenotype-related gene ranks using non-top-portion genes as positives

To maximize the efficiency of the propagation effect during the LBP process and rescue false negative genes from unlabeled genes, we selected the top-ranked 50 genes from DEG analysis and 50 genes from NP as positive genes for PU learning on the network. To assess the effectiveness of using top-ranked genes as input, we compared our approach with an alternative approach using secondary-top portion as input positive genes. For the secondary-top portion genes set preparation, we first selected the 51st–100th ranked DEGs from DEG analysis as positive genes. These genes, along with KO gene, were subsequently used as seed genes for NP. From the NP, we also retrieved the 51st–100th ranked genes as additional positive genes. Thus, the total number of secondary-top portion genes was the same as that of the top portion genes, but composed of genes ranked lower than the top portion genes.

Using top portion genes as input positive genes for PONYTA was more effective in most datasets compared to using secondary-top portion genes (Table 4). The capability of prioritizing phenotype-related genes was mostly degraded, including the dramatic case of Tcf4, where none of phenotype-related genes were prioritized within a top 200 range. However, for the Pax6 dataset, using secondary-top portion genes as positive genes achieved better performance for both $AUC_{200}$ and $AUC_{300}$ compared to the baseline approach. This discrepancy may be attributed to the fixed ranking criteria used for the DEG analysis output, where genes are ordered based on ascending adjusted *P*-values. This approach may overlook genes that are notably related to the Pax6 gene phenotype, which exhibit considerable alterations in log2FC values but fail to meet the criteria for significant adjusted *P*-values.

**Table 4.** Performance comparison using secondary-top portion as input positive genes. The higher partial AUC value is highlighted in bold.

Input positive genes	Nkx2-1 KO		Nr5a1 KO		Tcf4 KO		Pax6 KO		Sarm1 KO	
	$AUC_{200}$	$AUC_{300}$	$AUC_{200}$	$AUC_{300}$	$AUC_{200}$	$AUC_{300}$	$AUC_{200}$	$AUC_{300}$	$AUC_{200}$	$AUC_{300}$
Top portion	**0.0769**	**0.1179**	**0.1245**	**0.1853**	**0.1961**	**0.2496**	0.2988	0.3658	**0.1312**	**0.1900**
Secondary-top portion	0.0643	0.0780	0.0347	0.0561	0	0.0024	**0.4283**	**0.4700**	0.0238	0.0440

### 4.4 Evaluation of phenotype-related gene ranks with increased false positives

By using strict cutoff value, our approach aimed to achieve two objectives: first, to rescue false negative genes within unlabeled genes that were disregarded by strict cutoff values, and second, to mitigate the impact of false positive genes, which may introduce noise during PU learning. To demonstrate our approach’s performance considering these aspects, we evaluated our approach using positive genes with an increased rate of false positive genes. Instead of using only the top portion of genes, we harnessed a positive gene set ten times larger than the original approach, comprising the top 500 ranked genes from DEG analysis and NP, respectively. For studies yielding fewer than 500 genes in the DEG analysis output, such as Tcf4 and Sarm1, we utilized the gene list from DEG analysis prior to filtration based on their adjusted *P*-value and log2FC values.

Compared to the original approach employing a strict cutoff value, allowing an increased rate of false positive genes with a flexible cutoff value results in degraded performance across all datasets (Table 5). $AUC_{200}$ and $AUC_{300}$ values for all datasets degraded significantly. Interestingly, for Sarm1 datasets, where the DEG analysis output prior to filtration was utilized, almost none of the phenotype-related genes were captured within the highly-ranked portion. Given the higher likelihood of false positive genes within the unfiltered gene list from DEG analysis, the results suggest a potential flaw stemming from false positive genes during PU learning.

**Table 5.** Performance comparison using top portion with increased false positive genes rates. The higher partial AUC value is highlighted in bold.

Input positive genes	Nkx2-1 KO		Nr5a1 KO		Tcf4 KO		Pax6 KO		Sarm1 KO	
	$AUC_{200}$	$AUC_{300}$	$AUC_{200}$	$AUC_{300}$	$AUC_{200}$	$AUC_{300}$	$AUC_{200}$	$AUC_{300}$	$AUC_{200}$	$AUC_{300}$
Top portion	**0.0769**	**0.1179**	**0.1245**	**0.1853**	**0.1961**	**0.2496**	**0.2988**	**0.3658**	**0.1312**	**0.1900**
Increased FP	0.0371	0.0725	0.0606	0.0731	0.0941	0.1020	0.0645	0.1103	0	0.0127

### 4.5 Case study: functional analysis of prioritized genes

Our approach expects the rescued false negative genes, resulting from strict cutoff values, to be prominently ranked within the highly-ranked gene list. Therefore, to validate that the highly-ranked genes include a significant number of false negative genes related to phenotypic changes due to gene KO events, we performed gene set enrichment analysis (GSEA) on the top 300 genes from the obtained genes list for each phenotype-related genes dataset. The GSEA results for each dataset are shown for both the Gene Ontology (GO) Biological Process 2023 and BioPlanet 2019 databases.

#### 4.5.1 Tcf4

The transcription factor 4 (*Tcf4*) is crucial for neurodevelopment (Wittmann *et al.* 2021). Without the transcriptional regulatory role of the gene during forebrain development, neurodevelopmental disorders such as autism, schizophrenia and intellectual disability can occur. Alterations in gene expression patterns, including KO events, introduce disturbances in proper neocortical neuronal migration and the formation of dendrites and synapses (Li *et al.* 2019). At the molecular level, *Tcf4* affects the *Satb2* gene, a marker gene for interhemispheric projection neurons (IPNs) and is associated with the development of commissures and neuron projections.

**Figure 2.**
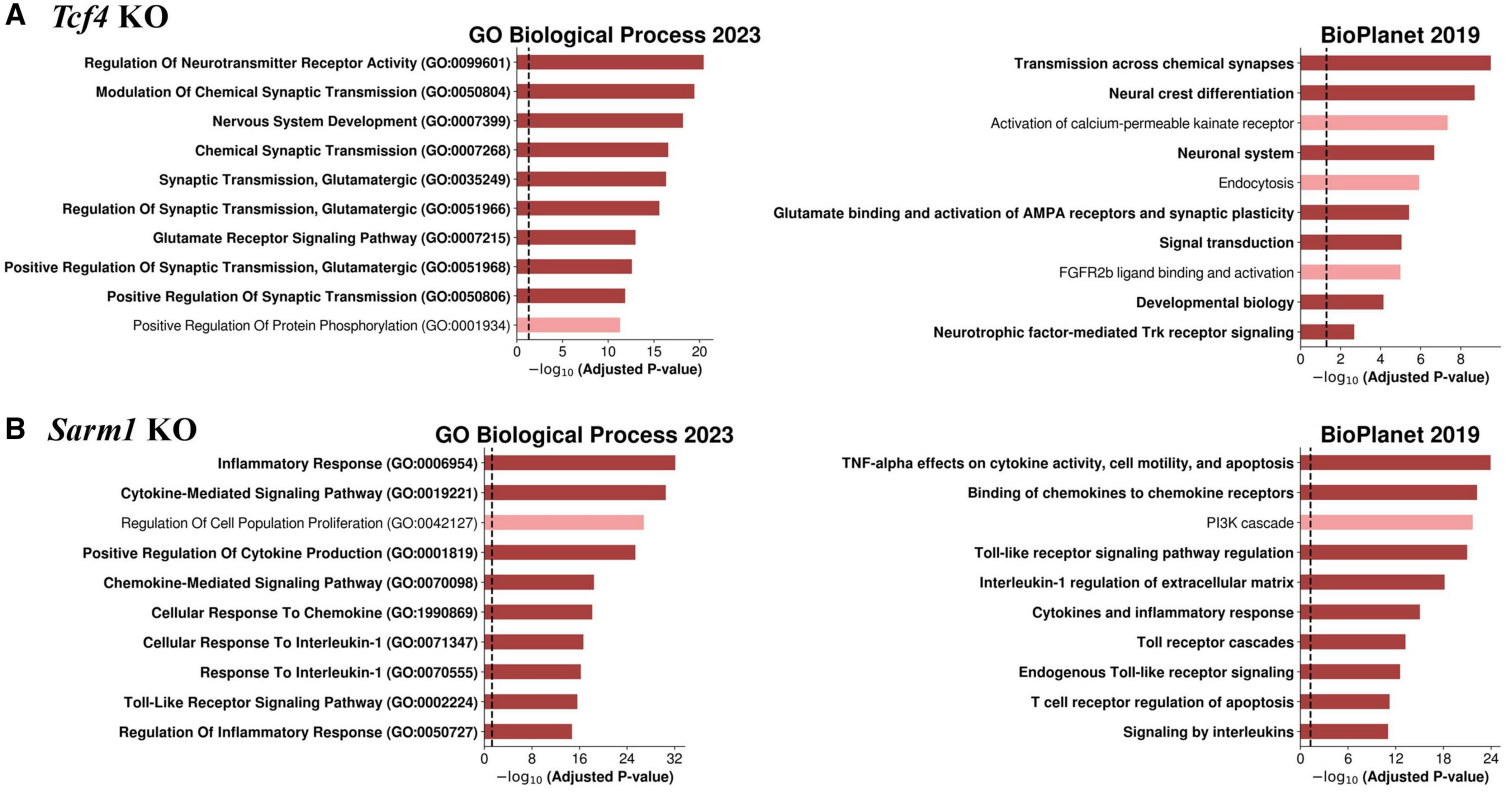
Enriched biological terms from GSEA on the top 300 prioritized genes from PONYTA for two databases: GO Biological Process 2023 and BioPlanet 2019. Terms associated with the known function of the KO gene are highlighted in bold, and their bar plots are accentuated. The dashed line on each plot indicates the position where the adjusted *P*-value is .05.

Through GSEA, we found that highly-ranked genes related to *Tcf4* are mainly associated with the nervous system, given that many terms were related with transmission between neurons and signal transduction (Fig. 2A). Given that IPNs are represented as glutamatergic neurons expressing *Satb2*, terms related to neuronal activity also include those for glutamatergic activity, indicating the functional relevance of highly-ranked genes to phenotypic changes.

#### 4.5.2 Sarm1

*Sarm1* prominently expressed in neurons and regulates cell death and axon degeneration under various conditions, such as glucose and oxygen deprivation, or oxidative stress (Doran *et al.* 2021). The degeneration of axons is attributed to the NADase activity in the toll-interleukin-1 receptor (TIR), activated when the inhibitory lock between TIR and Armadillo (ARM) domain is disrupted. Opposed to the role of the *Sarm1* gene in axon degradation, nicotinamide mononucleotide adenylyltransferase 2 (NMNAT2) prevents axon degradation (Figley and DiAntonio 2020). Once NMNAT2 is palmitoylated, a post-translational modification, it undergoes degradation process through MAPK pathway activation, ultimately allowing *Sarm1* to induce axon degeneration. In addition, upon neuron damage or inflammasome activation, *Sarm1* is responsible for the transcriptional regulation and production of cytokines and chemokines, including TNF, CCL7, CCL12, and CSF-1.

GSEA results for the prioritized genes showed terms related to cytokine and chemokine secretion with significant adjusted *P*-values (Fig. 2B). These terms align with the primary function of *Sarm1* in the axon degeneration process. Additionally, terms related to TLR regulation were observed, indicating that the activation of NADase activity, which leads to axon degeneration, is associated with highly-ranked genes. Considering that GO terms linked to prioritized genes exhibit a correlation with the phenotype observed in the *Sarm1* KO study with significant *P*-values, it can be asserted that PONYTA effectively rescues false negative genes from unlabeled genes. GSEA results for other datasets, *Nkx2–1*, *Nr5a1*, and *Pax6*, also show the relevance between GO terms and the phenotypes resulting from the KO event of each gene, as provided in the Supplementary Section of the article (Supplementary Fig. S1).

## 5 Discussion

Specifically by utilizing PU learning on graph, we aimed for flexible gene prioritization by focusing on the distribution of positive genes set by propagates their information within the biological graph as a pairwise Markov network. Given the inherent challenge of exhaustively labeling every gene as either directly or indirectly related to a specific KO phenotype, we harnessed PU learning to effectively handles the subset of positive genes. In particular, by propagating information across genes during PU learning on a biological network modeled as a pairwise Markov graph, we aimed for flexible gene prioritization using only a subset of labeled genes. By designating prioritized genes from both DEG analysis and NP as positive genes, our approach takes into account both gene expression patterns and cascade effects within the gene interaction network. This enables the prioritization of genes that are either explicitly or implicitly affected by gene ablation. Also, PONYTA rescues phenotype-related genes from being false negatives by prioritizing genes ranked above stringent cutoff values from two methods. This approach helps address the trade-off problem in setting cutoff values. Furthermore, by minimizing the number of top-ranked genes used as positive genes, we aimed to leverage key features of positive genes and mitigate the effect of false positives during the learning process. As a result, PONYTA enhances the identification of phenotype-related genes and rescues false negative genes more frequently within higher ranks compared to other gene prioritization methods, without heavily depending on set cutoff values for the aggregated gene ranked list. We suggest that the strength of PU learning has broad potential across various bioinformatics studies, including disease-related genes prioritization (Supplementary Table S19). Further exploration of gene prioritization using PU learning could be pursued with novel PU algorithms, such as Dist-PU (Zhao *et al.* 2022).

## Supplementary Material

btae634_Supplementary_Data

## Data Availability

The data underlying this article were obtained from the GEO database (GSE129584, GSE219271, GSE147247, and GSE182091) and the European Nucleotide Archive (PRJEB20579). For detailed information, refer to Table 1.
