# Supplementary material for: PONYTA: prioritization of phenotype-related genes from mouse KO events using PU learning on a biological network
Source: Bioinformatics. 2024 Oct 21;40(11):btae634. doi: 10.1093/bioinformatics/btae634 (PMC11561041; doi:10.1093/bioinformatics/btae634)
Supplement: btae634_Supplementary_Data [file btae634_supplementary_data.pdf]

PONYTA: prioritization of phenotype-related genes from mouse KO events using PU learning on a biological network

Jun Hyeong Kim, Bonil Koo and Sun Kim

Supplementary Information

Supplementary Information S1. Processing gene expression profile

To evaluate PONYTA across different type of studies, we gathered data from studies utilizing either single-cell RNA sequencing and bulk RNA sequencing from Gene Expression Omnibus (GEO) database. For single-cell RNA sequencing, we downloaded files with gene count values and processed them into a gene expression table format. We used the decoupleR (Badia-i Mompel et al., 2022) tool to annotate cell types and extract cells matching the cell types referenced in each study prior to additional data preparation steps. In the case of bulk RNA sequencing, raw sequencing files were downloaded in FASTQ format and trimmed using Trim Galore (Krueger, 2015). The trimmed reads were then mapped to the mm38 reference genome using STAR (Dobin et al., 2013). Finally, RSEM (Li and Dewey, 2011) was used to obtain the counts for each gene.

Supplementary Information S2. PU Learning on gene interaction network

To apply PU learning on a gene network, we employed GRAB (Yoo et al., 2021), a PU learning method designed for graph data $S = (G, E)$, where G is the set of nodes representing genes and E is the set of edge between genes. By interpreting the graph as a pairwise Markov network, GRAB captures the probabilistic connections between genes. This Markov network property allows GRAB to consider structure-based relationships between genes and enables effective information sharing through propagation between labeled and unlabeled genes. GRAB utilizes and updates an approximated prior value $\hat{\pi}_p$, which predicts the ratio of positive genes within the unlabeled genes, instead of relying on pre-defined constant value π_p. This minimizes the risk of misclassification by avoiding the use of improper prior values. GRAB consists of two main algorithms for PU learning on networks, the *marginalization step* and the *update step*:

Marginalization step

For an unlabeled gene i, a latent variable z_i indicates the probability of gene i belonging to the positive gene set. The joint probability of latent variables for all unlabeled genes can be marginalized utilizing the gene information itself and the edge information with their neighboring genes. Node potential g_i assigned to an unlabeled gene i implies the prior probability of the gene belonging to the positive gene set, independent of other genes. GRAB updates the approximate prior value $\hat{\pi}_p$ for every iteration, starting from an initial prior value of 0, assuming all unlabeled genes as negative. Within the gene interaction network, gene i has two node potential values: $g_i(+1)$, equal to the approximated prior $\hat{\pi}_p$, and $g_i(-1)$, with the sum of the two equal to 1. The edge potential e_{ij} implies the degree of homophily, ranging from 0 to 1, between genes i and j. The formula for the joint probability is expressed as:

$$p(\mathbf{z}|\mathbf{X}, \mathbf{y}) = \frac{1}{K} \prod_{i \in \mathcal{U}} g_i(z_i|\mathbf{X}) \prod_{(i,j) \in \mathcal{E}} e_{ij}(v_i, v_j|\mathbf{X}), \tag{S1}$$

where K indicates a normalization constant, $\mathcal{U}$ indicates the set of unlabeled genes, $\mathcal{E}$ indicates the set of edges between genes, $\mathbf{X}$ is the embedding matrix for all genes, $\mathbf{y}$ is the gene label vector for labeled genes, and v_i represents either $\mathbf{y_i}$ (if gene i is labeled) or $\mathbf{z_i}$ (if gene i is unlabeled). Using marginalized latent variable $\mathbf{z}$, the binary classifier for PU learning optimizes the objective function:

$$\begin{aligned}\mathcal{L}(\theta; \mathbf{X}, \mathbf{y}, \mathcal{R}, \mathcal{U}) =& \frac{1}{|\mathcal{R}|} \sum_{i \in \mathcal{R}} (-log \hat{y}_i(+1)) \\ &+ E_{\mathbf{z} \sim p(\mathbf{z}|\mathbf{X}, \mathbf{y})} [\frac{1}{|\mathcal{U}|} \sum_{j \in \mathcal{U}} (-log \hat{y}_j(z_j))]\end{aligned} \tag{S2}$$

minimizing the sum of negative log likelihood losses for labeled and unlabeled genes, where $\mathcal{R}$ stands for the set of positive labeled genes.

For large graphs, computation becomes extensive, making the learning process impractical. Therefore, instead of explicitly deriving expectation values, a pre-decomposed approximated joint distribution is used:

$$p(z|X,y) \approx \prod_i p_i(z_i|X,y). \tag{S3}$$

GRAB employs Loopy Belief Propagation (LBP) to get the approximated marginal distribution. During the propagation process, it updates the *messages* between every gene i and j:

$$m_{ij}(v) \leftarrow \sum_{u\in\{+1,-1\}} g_i(u)e_{ij}(u,v) \prod_{r\in H_i\setminus\{j\}} m_{ri}(u), \tag{S4}$$

which estimates the probability of gene j belonging to either positive or negative genes based on gene i, where H_i indicates the set of 1-hop neighbor genes of gene i. Once the messages values converge through LBP iteration, the approximated marginal probability value for each gene is calculated:

$$b_j(u) = \frac{g_j(u)\prod_{i\in H_j} m_{ij}(u)}{\sum_{v\in\{+1,-1\}} g_j(v)\prod_{i\in H_j} m_{ij}(v)}, \tag{S5}$$

indicating the belief values $b_j(u)$ of gene j for state u. Subsequently, the belief matrix B is constructed with calculated belief values for all genes.

Update step

The belief matrix subsequently used as a soft label for unlabeled genes during the *update step* to optimize parameter θ for the objective function $\tilde{\mathcal{L}}$:

$$\tilde{\mathcal{L}}(\theta;\mathbf{X},\mathbf{y},\mathbf{B},\mathcal{R},\mathcal{U}) = \frac{1}{|\mathcal{R}|}\sum_{i\in\mathcal{R}} l(\bar{\mathbf{y}}_\mathbf{i},\hat{\mathbf{y}}_\mathbf{i}) + \frac{1}{|\mathcal{U}|}\sum_{j\in\mathcal{U}} l(\mathbf{b}_\mathbf{j},\hat{\mathbf{y}}_\mathbf{j}) \tag{S6}$$

where $\bar{\mathbf{y}}_\mathbf{i}$ is the one-hot label vector for gene y_i within the positive genes set, and $\hat{\mathbf{y}}_\mathbf{j}$ is the probability vector for gene y_j belonging to the unlabeled genes set. The loss function l can vary; we specifically employed the negative log likelihood function.

Supplementary Information S3. Weighted DIBRA: Iterative distance-based aggregation method for gene rank aggregation

The aggregation process of weighted DIBRA starts by initializing the gene list A using the unweighted Borda Count method (Borda, 1781). The method then measure the distance between the aggregated gene rank list A and each individual gene rank list G^i from ith fold. By assigning weight values to items within the gene rank lists, with larger values for highly-ranked items and smaller values for lower-ranked items, the distance between two lists can be measured in terms of the cosine similarities of their item weight values. The distance from the aggregated gene list A is then employed to update the weight value assigned to each gene rank through the kernel function T:

$$\begin{gathered} G_w^{i,k} = G_w^{i,k-1} + T(d(G^i, A^{k-1})), \\ \text{where } T(d(G^i, A^{k-1})) = e^{-k\cdot d(G^i,A^{k-1})} \end{gathered} \tag{S7}$$

Here, $G_w^{i,k}$ is the weight assigned to gene rank G^i during kth weight update iteration, and d represents the distance function between two lists. As a result, weight values assigned to each gene rank list are updated with larger values, bounded by an upper bound value to prevent weight explosion, when their distance from the aggregated list A is smaller. The aggregated gene list A is also updated via Borda Count but in a weighted aggregation manner.

Since the crucial point of our approach lies in ranking phenotype-related genes within the highly-ranked portion of the aggregated gene list, genes distributed in the low ranked portion of each gene rank list are comparatively less important. Hence, once the iterations for weight update are completed, each gene rank list G is pruned to exclude genes within the low-rank portion prior to the final aggregation process. This is done by dropping genes with smaller weights compared to a cut-off value derived from their gene rank list weight assigned by equation (S7). The gene list obtained from the final aggregation process thereby exhibits refined quality for highly-ranked genes, being less affected by noise introduced by low-ranked genes.

Supplementary Information S4. Other metrics for prioritized gene rank evaluation

To further demonstrate the gene prioritization ability of PONYTA, we additionally computed the score using alternative metrics: Precision at Top-K, Recall at Top-K, F1-Score at Top-K, and Normalization Discounted Cumulative Gain (NDCG) (Järvelin and Kekäläinen, 2017).

- **Precision at Top-K (P@K)**

$$P@k = \frac{\text{Number of Relevant Items in Top } k}{k}$$

  - Measures the proportion of phenotype-related genes among the top-k ranked genes. A higher Precision at Top-K indicates a higher concentration of true positives among its top predictions.

- **Recall at Top-K (R@K)**

$$P@k = \frac{\text{Number of Relevant Items in Top } k}{\text{Total Number of Relevant Items}}$$

  - Measures the proportion of phenotype-related genes captured within the top-k ranked genes relative to the total number of phenotype-related genes. A higher Recall at Top-K indicates a larger portion of phenotype-related genes are among the top-k predictions.

- **F1-Score at Top-K (F1@K)**

$$P@k = \frac{2 \times P@k \times R@k}{P@K + R@k}$$

  - Measures the harmonic mean of Precision and Recall at Top-K. A higher F1-Score at Top-K indicates that both precision and recall are well-balanced.

- **Normalization Discounted Cumulative Gain (NDCG)**

$$nDCG@k = \frac{DCG@k}{IDCG@k}$$

$$DCG@k = \sum_{i=1}^{k} \frac{\text{rel}_i}{\log_2(i+1)}$$

  where:

  - rel_i: Relevance score of the item at rank i
  - DCG@k: Discounted Cumulative Gain at rank k
  - IDCG@k: Ideal Discounted Cumulative Gain at rank k

  - Measures the relevance of the prioritized gene list, emphasizing the importance of items ranked higher. A higher NDCG scores indicate better ranking of important genes at the top.

Supplementary Figures

Supplementary Figure S1. Gene Set Enrichment Analysis Plot

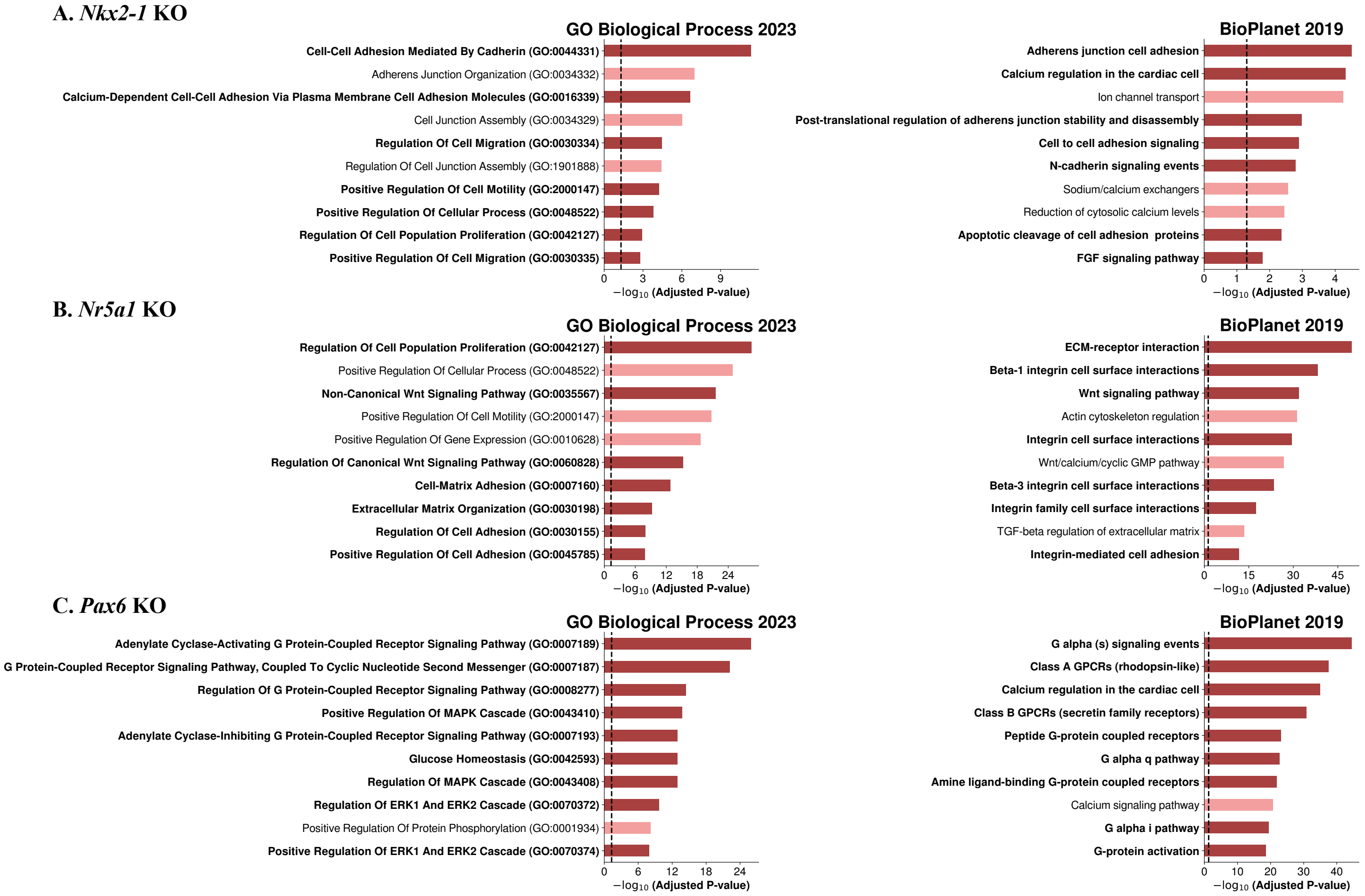

Supplementary Fig. S1: GSEA results for the top 300 prioritized genes from PONYTA for each gene KO study

Nkx2-1

The expression of NKX2-1, regulated by the fibroblast growth factor (FGF) signaling pathway, is a critical factor in alveolar type 1 (AT1) cells for alveologenesis (Serls et al., 2005; Yang et al., 2012). Dysfunction of the FGF signaling pathway by inhibiting the fibroblast growth factor receptor (FGFR) reduces NKX2-1 expression, consequently affecting lung specification and morphogenesis. When their morphogenesis function is disrupted, it leads to cell shrinkage, where cell shows elevated level of E-Cadherin (ECAD) at cell junctions, possibly due to protein redistribution. The gene is also associated with controlling the cell cycle by suppressing mitosis, leading to the quiescent property of AT1 cell (Little et al., 2019).

The main functions of *Nkx2-1* in AT1 cells aligned with GO terms from GSEA on the top 300 genes prioritized by PONYTA (Fig. S1A). The GSEA output involved GO terms associated with cell proliferation and cell adhesion, which align with the phenotypic results from *Nkx2-1* KO, suggesting the role of *Nkx2-1* in suppressing the proliferation of AT1 cells by inhibiting their mitosis. Considering the relevance to the term related to the FGF pathway, it can be inferred that the prioritized genes are also functionally associated with alveologenesis and morphogenesis pathways regulated by the *Nkx2-1* gene.

Nr5a1

The gene *Nr5a1* plays a crucial role in Sertoli cell (SC) functions, influencing cell proliferation, survival, and differentiation (Souali-Crespo et al., 2023). Upon the ablation of the *Nr5a1* gene within a cell, there is a sequential decline in SOX9 and COL-IV expression, which ultimately leads to testis cords disorganization due to basement membrane alteration. Disruption in the membrane causes dysregulation in integrin mediated cell adhesion and triggers anoikis, a form of cell death resulting from the detachment of cells from the extracellular matrix (ECM) (Taddei et al., 2012). Furthermore, SCs lacking the *Nr5a1* undergo molecular identity differentiation by adopting properties resembling fetal ovarian cells, evidenced by altered expression levels of *Wnt4* and *Fst* genes, indicative of the activation of the Wnt signaling pathway.

We conducted GSEA on the top 300 genes prioritized by our approach for the *Nr5a1* gene KO case (Fig. S1B). Given the significant role of *Nr5a1* in the cell adhesion process directly tied to cell fate, GSEA unveiled GO terms related to integrin-mediated cell adhesion and extracellular matrix organization with significant adjusted p-values, signifying that many genes mediating cell-adhesion are positioned within the highly-ranked genes. Moreover, the analysis implicated terms associated with the Wnt signaling pathway, suggesting that many genes prioritized by PU learning are involved in processes altering SC cell fate.

Pax6

The *Pax6* gene plays a crucial role in β cells, controlling glucose metabolism, and its disruption is associated with an increase in blood glucose levels (Mitchell et al., 2017). In more detail, under conditions of *Pax6* gene down-regulation, the G protein-coupled receptor 142 (Gpr14) in β cells is also down-regulated. This cascade process results in glucose tolerance and makes cells susceptible to exhibiting a diabetic phenotype (Al-Amily et al., 2019; Gosmain et al., 2012). When glucose levels are elevated, the ERK and p38 MAPK pathways are also known to be upregulated, although the precise mechanisms leading to their activation are yet to be established (Delobel and Dalle, 2021). Additionally, *Pax6* regulates insulin secretion by modulating G protein-coupled receptors (GPCRs), aligning with its role in regulating glucose levels (Balakrishnan et al., 2014; Evans et al., 2003).

Given the significance of GPCRs in relation to the regulatory role of *Pax6* with glucose levels, the terms associated with GPCRs are featured as significant functional pathways among the highly-ranked genes identified through PU learning (Fig. S1C). Additionally, ERK and MAPK pathways related GO terms exhibit significant p-values, emphasizing the functional involvement of prioritized genes within the regulatory functions governed by the *Pax6* gene. This concurrence further substantiates that the identified genes play pivotal roles in pathways linked to *Pax6* and its regulatory functions in glucose metabolism.

Supplementary Tables

Supplementary Tables S1-5. Affected genes for each study on gene knock-out

- scRNA-Seq

1. *Nkx2-1*

Supplementary Table S1: *Nkx2-1* KO Phenotype-related genes

Mki67	Cym	Tff2	Ctse	Hnf4a
Vsig1	Pigr	Rtkn2	Wnt3a	Hopx
Col4a3	Ager	Scnn1g	Sema3a	Clic5
Ifit3	Wnt7a	Msn	Vegfa	Samhd1
Pmp22	Akap5	Timp3	Aqp5	Qk
Emp2	Cav1	Ahnak	Igfbp7	Pdpn
Lmo7	Cavin2	Tspan8	Igfbp2	Hopx
Ager	Pdpn	Aqp5		

2. *Nr5a1*

Supplementary Table S2: *Nr5a1* KO Phenotype-related genes

Amh	Sox9	Sox8	Trp53	Stra8
Rec8	Nanos2	Piwil4	Fst	Rspo1
Wnt4	Dmrt1	Gja1	Jam2	Ptk2b
Itga6	Col4a1	Col4a2	Col3a1	Col5a2
Col6a6	Fn1	Fbln1	Mdk	Wnt4
Hk1	Aldoa	Pgam1	Enol	Nr2f2
Foxl2	Ptk2b	Coll8a1	Fn1	Nid2
Dcn	Hsd17b1	Hsd17b3	Fgf9	Fgfr1
Cyp26b1	Dhh	Pdgfa	Wnt5a	Gpi1
Aldoc	Pgk1	Eno3	Pkm	Ldhb

3. *Tcf4*

Supplementary Table S3: *Tcf4* KO Phenotype-related genes

Satb2	Gap43	Gfap	Satb2	Cd24a
Gpm6a	Neurod2	Ptn	Bcl11a	Fezf2
Hnrnpk	Id2	Dcx	Epha5	Nfib
Tubb3	Ctcf	Foxg1	Smarca4	Cux1
Pou3f3	Sox11	Sox5	Foxp2	Foxg1
Plxna2				

- Bulk RNA-Seq

1. *Pax6*

Supplementary Table S4: *Pax6* KO Phenotype-related genes

Ins2	Slc30a8	Mafa	Slc2a2	G6pc2
Glp1r	Slc16a1	Pdx1	Nkx6-1	Pcsk1
Ins1	Gjd2	Gcg	Ghrl	Gck
Slc16a1	Ldha	Pdgfra	Cxcl12	Igfbp4
Oat	Ppy	Sst	Pcsk2	Ero1lb
G6pc2	Kcnj5	Gjd2	Rfx6	

2. *Sarm1*

Supplementary Table S5: *Sarm1* KO Phenotype-related genes

Ccl2	Ccl7	Ccl12	Csf1	Ccl5
Tnf	Il6	S100a8	Ctsk	Depdc1a
Aldh1l1	Folr2	Hyal1	Cx3cr1	Tnfsf13
Plscr3	Acap1	Xaf1	Ccl9	Ccl6
Ly6c2	Olfr111	Ms4a14	Acap1	Tfrc
Rps29	Rpl38	Ndufb3	Atp5k	Xaf1

Supplementary Table S6. Phenotype induced by the gene KO event refers from the KO studies

Supplementary Table S6: Phenotype induced by the gene KO event refers from the KO studies.

KO Gene	Phenotype induced by KO event
Nkx2-1	Loss of alveolar type 1 (AT1) cell characteristics, including molecular markers, morphology, and quiescence, leading to alveolar simplification and lethality
Nr5a1	Impaired Sertoli cell identity, increased cell death, testis cord disorganization, and germ cell meiosis
Tcf4	Impaired neurodevelopment processes, affecting interhemispheric connectivity and commissure formation, and associated with neurodevelopmental disorders
Pax6	Progressive hyperglycemia, reduced insulin content and secretion, impaired glucose sensing, and disrupted pancreatic b-cell function
Sarm1	Impaired axonal degeneration in response to neurotoxic conditions, such as oxygen deprivation or oxidative stress

Supplementary Tables S7-10. Phenotype-related genes prioritization evaluation with various metrics

Supplementary Table S7: Performance comparison based on Precision at Top-K (P@K) values for each method across different datasets.

Method	Nkx2-1 KO		Nr5a1 KO		Tcf4 KO		Pax6 KO		Sarm1 KO	
	P@200	P@300	P@200	P@300	P@200	P@300	P@200	P@300	P@200	P@300
Node2vec	0.0050	0.0033	0.0150	0.0133	0.0100	0.0067	0.0050	0.0067	0.0050	**0.0133**
DIAMOnD	0	0	0.0025	0.0233	0.0050	0.0033	0.0050	0.0067	0.0100	0.0100
RWR	0.0100	0.0100	0.0300	0.0333	0.0050	**0.0267**	0.0050	0.0067	0.0050	0.0033
GenePanda	0.0050	0.0100	0.0050	0.0067	0	0	0.0100	0.0067	0	0
PONYTA	**0.0200**	**0.0267**	**0.0550**	**0.0400**	**0.0300**	0.0233	**0.0400**	**0.0267**	**0.0200**	**0.0133**

Supplementary Table S8: Performance comparison based on Recall at Top-K (R@K) values for each method across different datasets.

Method	Nkx2-1 KO		Nr5a1 KO		Tcf4 KO		Pax6 KO		Sarm1 KO	
	R@200	R@300	R@200	R@300	R@200	R@300	R@200	R@300	R@200	R@300
Node2vec	0.0345	0.0345	0.0769	0.1026	0.1053	0.1053	0.0625	0.1250	0.0769	**0.3077**
DIAMOnD	0	0	0.1282	0.1795	0.0526	0.0526	0.0625	0.1250	0.1538	0.2308
RWR	0.0690	0.1034	0.1538	0.2564	**0.3158**	**0.4211**	0.0625	0.1250	0.0769	0.0769
GenePanda	0.0345	0.1034	0.0256	0.0513	0	0	0.1250	0.1250	0	0
PONYTA	**0.1379**	**0.2759**	**0.2821**	**0.3077**	**0.3158**	0.3684	**0.5000**	**0.5000**	**0.3077**	**0.3077**

Supplementary Table S9: Performance comparison based on F1-Score at Top-K values for each method across different datasets.

Method	Nkx2-1 KO		Nr5a1 KO		Tcf4 KO		Pax6 KO		Sarm1 KO	
	$F1$@200	$F1$@300	$F1$@200	$F1$@300	$F1$@200	$F1$@300	$F1$@200	$F1$@300	$F1$@200	$F1$@300
Node2vec	0.0090	0.0060	0.0250	0.0240	0.0180	0.0130	0.0090	0.0130	0.0090	**0.0260**
DIAMOnD	0	0	0.0420	0.0410	0.0090	0.0060	0.0090	0.0130	0.0190	0.0190
RWR	0.0170	0.0180	0.0500	0.0590	**0.0550**	**0.0500**	0.0090	0.0130	0.0090	0.0060
GenePanda	0.0090	0.0180	0.0080	0.0120	0	0	0.0190	0.0130	0	0
PONYTA	**0.0350**	**0.0490**	**0.0920**	**0.0710**	**0.0550**	0.0440	**0.0740**	**0.0510**	**0.0380**	**0.0260**

Supplementary Table S10: Performance comparison based on Normalized Discounted Cumulative Gain (NDCG) values for each method across different datasets.

Method	Nkx2-1 KO		Nr5a1 KO		Tcf4 KO		Pax6 KO		Sarm1 KO	
	$NDCG_{200}$	$NDCG_{200}$	$NDCG_{200}$	$NDCG_{200}$	$NDCG_{200}$	$NDCG_{200}$	$NDCG_{200}$	$NDCG_{200}$	$NDCG_{200}$	$NDCG_{200}$
Node2vec	0.5743	0.6463	0.5361	0.6134	**0.6916**	<u>0.7487</u>	0.6643	0.7342	<u>0.6990</u>	**0.7763**
DIAMOnD	0.5643	0.6380	0.5535	0.6327	0.6421	0.7084	0.6644	<u>0.7345</u>	0.6920	0.7649
RWR	<u>0.5786</u>	<u>0.6549</u>	<u>0.5703</u>	<u>0.6566</u>	0.6815	**0.7516**	0.6629	0.7329	0.6937	0.7543
GenePanda	0.5711	0.6538	0.5217	0.6015	0.6301	0.6986	<u>0.6703</u>	0.7333	0.6843	0.7468
PONYTA	**0.5974**	**0.6856**	**0.5934**	**0.6620**	<u>0.6840</u>	0.7481	**0.7329**	**0.7840**	**0.7109**	<u>0.7743</u>

Supplementary Tables S11-15. Gene Rank for all phenotype-related genes

Each supplementary table presents ranking information for phenotype-related genes from various gene prioritization algorithms. log2FC and adj. p-value columns display the log2FoldChange values and adjusted p-value of each gene from the DEG analysis, while NP Rank and NP Score columns indicate the network propagation rank and score values of each gene, respectively. For the case in which either a gene or value is not included in the output list of the corresponding method, it is indicated as '-'.

Benchmark models

- Randomwalk with restart (RWR) Köhler et al. (2008).
- Node2vec (N2V) Grover and Leskovec (2016)
- DIAMOnD Ghiassian et al. (2015)
- GenePanda Yin et al. (2017)

Supplementary Table S11. Nkx2-1 KO

Supplementary Table S11: *Nkx2-1* KO phenotype-related gene ranks in models

Affected Gene	PONYTA	RWR	N2V	Genepanda	DIAMOnD	DEG Rank	log2FC	adj.p-value	NP Rank	NP Score
Ahnak	18	246	-	510	-	-	-0.533	1.325e-20	561	0.0136
Cavin2	73	-	-	595	-	-	-	-	3190	0.0039
Hopx	115	132	-	-	-	149	1.544	6.651e-40	349	0.0176
Pdpn	158	98	-	525	-	-	0.171	1.000	436	0.0154
Vegfa	234	-	-	-	323	-	0.786	7.564e-14	370	0.0171
Tspan8	273	-	-	518	-	-	0.784	5.788e-24	7979	0.0010
Cym	299	-	-	-	-	963	-8.637	3.902e-06	3713	0.0033
Igfbp2	416	-	-	-	-	300	1.514	9.175e-26	7654	0.0011
Mki67	424	-	-	-	384	2017	-1.001	1.000	433	0.0156
Igfbp7	633	-	-	-	-	-	-0.158	1.000	8824	0.0008
Clic5	828	-	-	-	-	-	-	-	2411	0.0049
Akap5	890	-	-	-	-	-	-0.380	9.794e-09	5993	0.0018
Msn	955	-	28	-	-	-	0.681	6.210e-19	730	0.0118
Lmo7	1324	-	-	-	-	-	-0.673	7.167e-33	8747	0.0008
Tff2	1326	-	-	218	-	843	-6.089	8.256e-08	861	0.0109
Scnn1g	1410	-	-	312	-	186	1.751	1.020e-35	833	0.0111
Timp3	-	-	-	-	-	-	0.444	1.635e-08	3259	0.0038
Sema3a	-	-	-	-	-	560	1.267	3.355e-14	1034	0.0097
Col4a3	-	-	-	-	-	220	1.322	2.588e-32	3471	0.0036
Hnf4a	-	-	-	-	-	1155	-5.073	8.430e-04	2105	0.0055
Pmp22	-	-	-	286	-	122	1.572	3.698e-45	2869	0.0043
Wnt3a	-	-	-	142	-	372	2.595	5.195e-21	1530	0.0073
Wnt7a	-	-	-	-	-	1451	1.142	2.023e-01	4444	0.0027
Pigr	-	-	-	-	-	-	-	-	7621	0.0011
Ctse	-	-	-	-	-	937	-5.309	1.807e-06	13001	0.0001
Qk	-	-	-	-	-	-	0.785	5.211e-13	5574	0.0020
Ifit3	-	-	-	-	-	381	4.164	1.774e-20	2871	0.0043
Samhd1	-	-	-	-	-	139	1.751	6.080e-42	9341	0.0006

Supplementary Table S12. Nr5a1 KO

Supplementary Table S12: *Nr5a1* KO phenotype-related gene ranks in models

Affected Gene	PONYTA	RWR	N2V	Genepanda	DIAMOnD	DEG Rank	log2FC	adj.p-value	NP Rank	NP Score
Dcn	13	246	94	437	193	427	-3.474	2.176e-27	169	0.0305
Fgf9	38	-	98	495	-	-	-0.711	6.977e-01	758	0.0119
Pdgfa	46	231	-	549	297	131	2.952	6.586e-58	447	0.0176
Sox9	139	247	-	521	-	297	1.266	2.225e-36	221	0.0264
Col4a1	147	58	344	499	69	363	2.011	4.393e-31	395	0.0189
Itga6	169	233	321	147	-	234	1.176	7.850e-43	215	0.0269
Rspo1	179	-	-	448	-	188	-3.930	4.501e-49	1526	0.0075
Col6a6	182	-	-	340	19	75	-4.890	1.195e-72	3114	0.0040
Mdk	189	-	-	-	-	94	-1.827	5.591e-67	981	0.0103
Fgfr1	215	36	-	-	280	468	-2.814	4.052e-25	398	0.0188
Col4a2	314	-	291	523	39	664	1.676	1.693e-18	551	0.0150
Foxl2	346	76	-	240	-	1422	-5.899	3.177e-06	496	0.0162
Dmrt1	363	-	-	-	-	-	0.505	1.000	847	0.0112
Nid2	534	-	-	-	-	118	-4.175	3.415e-60	1869	0.0063
Col5a2	539	-	342	316	-	422	-3.076	1.137e-27	552	0.0150
Fbln1	726	-	-	461	-	341	-2.513	5.846e-33	3802	0.0033
Dhh	797	127	80	-	-	273	1.229	5.129e-38	271	0.0231
Gja1	1138	-	-	-	-	74	2.915	1.163e-72	1103	0.0095
Ptk2b	1501	-	-	-	147	859	1.118	5.264e-14	1443	0.0078
Hsd17b1	-	-	-	-	-	106	3.600	3.384e-63	2049	0.0059
Jam2	-	-	-	-	-	151	2.845	3.346e-54	6942	0.0014
Hk1	-	4	-	-	-	968	2.289	3.791e-12	545	0.0151
Pgk1	-	-	-	-	-	-	-0.952	9.280e-26	3226	0.0039
Pkm	-	-	-	-	-	-	-0.458	2.199e-13	1042	0.0098
Cyp26b1	-	-	-	-	-	-	0.900	1.224e-04	1203	0.0089
Eno3	-	-	-	-	-	743	-1.567	8.783e-17	3333	0.0037
Aldoa	-	-	-	-	-	183	1.145	1.971e-49	3145	0.0039
Aldoc	-	-	-	-	-	1143	-1.733	2.096e-09	3307	0.0038
Stra8	-	-	-	-	-	1099	-4.929	5.324e-10	3059	0.0041
Pgam1	-	-	-	-	-	-	0.555	2.540e-23	5326	0.0022
Gpi1	-	-	-	-	-	-	-0.272	1.000	1981	0.0060
Nanos2	-	-	-	-	-	-	-	-	6148	0.0017
Sox8	-	-	-	-	-	711	1.838	2.993e-17	11486	0.0003
Ldhb	-	-	-	-	-	-	-0.373	2.747e-17	3924	0.0032
Rec8	-	-	-	-	-	1129	-3.044	1.268e-09	3864	0.0032
Piwil4	-	-	-	-	-	-	-	-	9562	0.0006

Supplementary Table S13. Tcf4 KO

Supplementary Table S13: *Tcf4* KO phenotype-related gene ranks in models

Affected Gene	PONYTA	RWR	N2V	Genepanda	DIAMOnD	DEG Rank	log2FC	adj.p-value	NP Rank	NP Score
Sox5	31	195	-	349	-	-	-	-	461	0.0156
Fezf2	60	119	-	565	-	-	-0.930	7.763e-02	1141	0.0090
Satb2	61	234	-	425	-	-	-0.596	7.949e-06	596	0.0132
Neurod2	100	106	-	480	-	-	-0.243	1.000	2231	0.0057
Cux1	165	-	-	476	-	-	0.770	3.811e-11	3463	0.0039
Dcx	229	17	-	488	-	-	0.779	1.037e-41	796	0.0110
Foxp2	437	-	-	310	-	-	0.337	1.000	2579	0.0051
Bcl11a	497	196	-	-	-	-	0.282	1.078e-01	433	0.0162
Gap43	543	-	-	-	22	-	-0.275	2.725e-03	2410	0.0054
Id2	695	98	-	-	-	-	0.324	4.383e-03	665	0.0123
Sox11	932	-	-	-	-	-	-0.149	1.510e-11	8861	0.0008
Smarca4	1100	-	1	-	-	-	0.687	1.551e-08	459	0.0156
Cd24a	-	-	-	-	-	-	-	-	2922	0.0045
Hnrnpk	-	-	188	-	-	-	0.329	3.343e-17	659	0.0124
Ctcf	-	-	-	-	-	-	0.475	3.781e-01	3046	0.0043
Ptn	-	-	-	-	-	-	-0.484	2.047e-12	4949	0.0025
Pou3f3	-	-	-	-	-	-	-0.233	1.000	10311	0.0004
Plxna2	-	-	-	-	-	-	0.758	4.051e-10	4390	0.0029

Supplementary Table S14. Pax6 KO

Supplementary Table S14: *Pax6* KO phenotype-related gene ranks in models

Affected Gene	PONYTA	RWR	N2V	Genepanda	DIAMOnD	DEG Rank	log2FC	adj.p-value	NP Rank	NP Score
Nkx6-1	48	-	-	173	-	-	0.771	3.519e-09	710	0.0130
Ppy	67	-	-	395	-	-	-0.222	7.329e-01	558	0.0148
Pcsk1	86	-	-	188	-	-	0.554	1.136e-03	1753	0.0070
Pcsk2	142	-	-	-	-	-	0.724	1.267e-18	4260	0.0029
Rfx6	178	-	-	-	-	72	-1.522	3.518e-58	2660	0.0049
Pdgfra	426	-	-	-	70	-	-0.778	6.452e-01	335	0.0192
Igfbp4	858	-	-	-	-	-	-0.565	6.334e-01	10244	0.0005
Slc16a1	1373	168	-	-	-	-	-0.487	5.090e-01	1822	0.0067
Cxcl12	-	-	-	-	214	-	-0.777	5.255e-01	658	0.0136
Ldha	-	-	-	-	-	-	-0.326	7.348e-01	303	0.0200
Oat	-	-	-	-	-	-	-0.552	4.668e-01	3258	0.0040
Ero1lb	-	-	-	-	-	-	-	-	6877	0.0014
Gjd2	-	-	-	-	-	-	-0.758	7.298e-07	7299	0.0013

Supplementary Table S15. Sarm1 KO

Supplementary Table S15: *Sarm1* KO phenotype-related gene ranks in models

Affected Gene	PONYTA	RWR	N2V	Genepanda	DIAMOnD	DEG Rank	log2FC	adj.p-value	NP Rank	NP Score
Csf1	50	304	15	519	-	-	-0.801	2.105e-01	348	0.0196
Cx3cr1	85	302	312	498	192	212	1.292	1.024e-01	169	0.0296
Ccl7	148	-	232	591	186	-	-0.204	4.325e-01	601	0.0136
Ccl12	319	75	216	-	281	-	-0.234	7.519e-01	388	0.0185
Tfrc	-	-	-	-	-	-	0.296	3.062e-01	1039	0.0091
Aldh1l1	-	-	-	-	-	-	-0.957	3.129e-06	7838	0.0011
Folr2	-	-	-	-	-	-	-0.116	9.340e-01	11137	0.0004
Ndufb3	-	-	-	-	-	-	0.046	9.051e-01	2788	0.0042
Depdc1a	-	-	-	-	-	-	-0.350	7.644e-01	8391	0.0010
Atp5k	-	-	-	-	-	-	0.074	7.697e-01	3559	0.0033
Rps29	-	-	-	-	-	-	0.252	4.328e-01	1464	0.0072
Rpl38	-	-	-	-	-	-	0.181	6.267e-01	1883	0.0059

Supplementary Tables S16-18. Phenotype-related genes rank evaluation with various gene rank aggregation method

For robustness evaluation using alternative aggregation methods, we computed partial AUC values on gene rank list obtained from three different gene rank aggregation methods, using the top 50 DEGs and 100 NP genes as input.

- Median rank aggregation (Fagin et al., 2003).
- Linear combination with Borda normalization (Renda and Straccia, 2003)
- Majoritarian method (Farah and Vanderpooten, 2007)

Supplementary Table S16: Performance comparison based on partial AUC values using median rank aggregation as aggregation method. The highest partial AUC value for each case is indicated in bold, and the second highest partial AUC value is underlined.

Method	Nkx2-1 KO		Nr5a1 KO		Tcf4 KO		Pax6 KO		Sarm1 KO	
	AUC_{200}	AUC_{300}	AUC_{200}	AUC_{300}	AUC_{200}	AUC_{300}	AUC_{200}	AUC_{300}	AUC_{200}	AUC_{300}
Node2vec	0	0.0040	0.0629	0.0790	0	0.0156	0	0	0.1346	0.1731
DIAMOnD	0	0.0092	0.0686	0.0920	0	0	0.0262	0.0451	0.1100	0.1567
RWR	0.0362	0.0813	0.0950	0.1354	0.0817	0.1598	<u>0.0927</u>	<u>0.1172</u>	0.1271	0.1681
GenePanda	<u>0.0539</u>	<u>0.0863</u>	**0.1501**	**0.1827**	<u>0.1053</u>	<u>0.1628</u>	0.0715	0.1000	**0.2062**	**0.2208**
PONYTA	**0.1011**	**0.1515**	<u>0.1011</u>	<u>0.1745</u>	**0.1108**	**0.1993**	**0.2623**	**0.3131**	<u>0.1496</u>	<u>0.1875</u>

Supplementary Table S17: Performance comparison based on partial AUC values using linear combination with borda normalization as aggregation method. The highest partial AUC value for each case is indicated in bold, and the second highest partial AUC value is underlined.

Method	Nkx2-1 KO		Nr5a1 KO		Tcf4 KO		Pax6 KO		Sarm1 KO	
	AUC_{200}	AUC_{300}	AUC_{200}	AUC_{300}	AUC_{200}	AUC_{300}	AUC_{200}	AUC_{300}	AUC_{200}	AUC_{300}
Node2vec	0	0.0106	0.0851	0.1001	0	0.0081	0	0	<u>0.1879</u>	0.2150
DIAMOnD	0	0.0127	0.0747	0.0961	0	0	0.0338	0.0554	0.1233	0.1656
RWR	0.0500	<u>0.0915</u>	0.0957	0.1427	0.0572	0.1443	0.0700	<u>0.1064</u>	0.1508	0.1839
GenePanda	<u>0.0545</u>	0.0857	**0.1507**	**0.1817**	<u>0.1044</u>	<u>0.1587</u>	<u>0.0712</u>	0.0987	**0.2058**	<u>0.2206</u>
PONYTA	**0.0612**	**0.1175**	<u>0.1135</u>	<u>0.1810</u>	**0.0919**	**0.1844**	**0.2219**	**0.2887**	0.1717	**0.2247**

Supplementary Table S18: Performance comparison based on partial AUC values using Majoritarian method as aggregation method. The highest partial AUC value for each case is indicated in bold, and the second highest partial AUC value is underlined.

Method	Nkx2-1 KO		Nr5a1 KO		Tcf4 KO		Pax6 KO		Sarm1 KO	
	AUC_{200}	AUC_{300}	AUC_{200}	AUC_{300}	AUC_{200}	AUC_{300}	AUC_{200}	AUC_{300}	AUC_{200}	AUC_{300}
Node2vec	0	0.0042	0.0608	0.0776	0	0.0163	0	0	0.1338	0.1725
DIAMOnD	0	0.0089	0.0686	0.0920	0	0	0.0262	0.0451	0.1100	0.1567
RWR	0.0357	0.0800	0.0960	0.1388	0.0797	0.1589	<u>0.0885</u>	<u>0.1164</u>	0.1275	0.1683
GenePanda	<u>0.0530</u>	<u>0.0869</u>	**0.1506**	**0.1815**	<u>0.1036</u>	<u>0.1606</u>	0.0727	0.1021	**0.2058**	**0.2206**
PONYTA	**0.1018**	**0.1577**	<u>0.0971</u>	<u>0.1689</u>	**0.1194**	**0.2035**	**0.2669**	**0.3272**	<u>0.1438</u>	<u>0.1928</u>

Supplementary Tables S19. Disease-related genes prioritization evaluation

To further assess PONYTA's performance beyond phenotype-related genes prioritization, we evaluated its ability to prioritize disease-related genes. We obtained DEG analysis results for two disease, psoriasis and influenza, from DiSignAtlas (Zhai et al., 2024). Using gene sets composed of top-ranked DEGs combined with top-ranked NP genes, where DEGs served as seed genes, we evaluated PONYTA's ability to prioritize disease-related genes from the Mouse Genome Informatics (MGI, www.informatics.jax.org) database. As with our previous KO study evaluation, we calculated partial AUC values for two different top-gene sets to measure performance.

Supplementary Table S19: Disease-related genes prioritization ability comparison based on partial AUC values.

Model	Psoriasis		Influenza	
	AUC_{200}	AUC_{300}	AUC_{200}	AUC_{300}
Node2vec	0	0	0.0209	0.0498
DIAMOnD	0.0017	0.0322	0.0776	0.1030
RWR	0.0396	0.0635	0.0405	0.0583
GenePanda	0.0276	0.0419	0.0351	0.0707
PONYTA	**0.0469**	**0.0683**	**0.1229**	**0.1521**

References

Al-Amily, I. M., Dunér, P., Groop, L., and Salehi, A. (2019). The functional impact of g protein-coupled receptor 142 (gpr142) on pancreatic β-cell in rodent. *Pflügers Archiv-European Journal of Physiology*, 471:633–645.

Badia-i Mompel, P., Vélez Santiago, J., Braunger, J., Geiss, C., Dimitrov, D., Müller-Dott, S., Taus, P., Dugourd, A., Holland, C. H., Ramirez Flores, R. O., et al. (2022). decoupler: ensemble of computational methods to infer biological activities from omics data. *Bioinformatics Advances*, 2(1):vbac016.

Balakrishnan, S., Sadasivam, M., Kannan, A., Panneerselvam, A., and Prahalathan, C. (2014). Glucose modulates pax6 expression through the jnk/p38 map kinase pathway in pancreatic beta-cells. *Life sciences*, 109(1):1–7.

Borda, J. d. (1781). M'emoire sur les' elections au scrutin. *Histoire de l'Acad'emie Royale des Sciences*.

Delobel, M. and Dalle, S. (2021). G-protein–coupled receptors controlling pancreatic β-cell functional mass for the treatment of type 2 diabetes. *Current Opinion in Endocrine and Metabolic Research*, 16:113–118.

Dobin, A., Davis, C. A., Schlesinger, F., Drenkow, J., Zaleski, C., Jha, S., Batut, P., Chaisson, M., and Gingeras, T. R. (2013). Star: ultrafast universal rna-seq aligner. *Bioinformatics*, 29(1):15–21.

Evans, J. L., Goldfine, I. D., Maddux, B. A., and Grodsky, G. M. (2003). Are oxidative stress- activated signaling pathways mediators of insulin resistance and β-cell dysfunction? *Diabetes*, 52(1):1–8.

Fagin, R., Kumar, R., and Sivakumar, D. (2003). Efficient similarity search and classification via rank aggregation. In *Proceedings of the 2003 ACM SIGMOD international conference on Management of data*, pages 301–312.

Farah, M. and Vanderpooten, D. (2007). An outranking approach for rank aggregation in information retrieval. In *Proceedings of the 30th annual international ACM SIGIR conference on Research and development in information retrieval*, pages 591–598.

Ghiassian, S. D., Menche, J., and Barabási, A.-L. (2015). A disease module detection (diamond) algorithm derived from a systematic analysis of connectivity patterns of disease proteins in the human interactome. *PLoS computational biology*, 11(4):e1004120.

Gosmain, Y., Katz, L. S., Masson, M. H., Cheyssac, C., Poisson, C., and Philippe, J. (2012). Pax6 is crucial for β-cell function, insulin biosynthesis, and glucose-induced insulin secretion. *Molecular endocrinology*, 26(4):696–709.

Grover, A. and Leskovec, J. (2016). node2vec: Scalable feature learning for networks. In *Proceedings of the 22nd ACM SIGKDD international conference on Knowledge discovery and data mining*, pages 855–864.

Järvelin, K. and Kekäläinen, J. (2017). Ir evaluation methods for retrieving highly relevant documents. In *ACM SIGIR Forum*, volume 51, pages 243–250. ACM New York, NY, USA.

Köhler, S., Bauer, S., Horn, D., and Robinson, P. N. (2008). Walking the interactome for prioritization of candidate disease genes. *The American Journal of Human Genetics*, 82(4):949–958.

Krueger, F. (2015). Trim galore!: A wrapper around cutadapt and fastqc to consistently apply adapter and quality trimming to fastq files, with extra functionality for rrbs data. *Babraham Institute*.

Li, B. and Dewey, C. N. (2011). Rsem: accurate transcript quantification from rna-seq data with or without a reference genome. *BMC bioinformatics*, 12:1–16.

Little, D. R., Gerner-Mauro, K. N., Flodby, P., Crandall, E. D., Borok, Z., Akiyama, H., Kimura, S., Ostrin, E. J., and Chen, J. (2019). Transcriptional control of lung alveolar type 1 cell development and maintenance by nk homeobox 2-1. *Proceedings of the National Academy of Sciences*, 116(41):20545–20555.

Mitchell, R. K., Nguyen-Tu, M.-S., Chabosseau, P., Callingham, R. M., Pullen, T. J., Cheung, R., Leclerc, I., Hodson, D. J., and Rutter, G. A. (2017). The transcription factor pax6 is required for pancreatic β cell identity, glucose-regulated atp synthesis, and ca2+ dynamics in adult mice. *Journal of Biological Chemistry*, 292(21):8892–8906.

Renda, M. E. and Straccia, U. (2003). Web metasearch: rank vs. score based rank aggregation methods. In *Proceedings of the 2003 ACM symposium on Applied computing*, pages 841–846.

Serls, A. E., Doherty, S., Parvatiyar, P., Wells, J. M., and Deutsch, G. H. (2005). Different thresholds of fibroblast growth factors pattern the ventral foregut into liver and lung.

Souali-Crespo, S., Condrea, D., Vernet, N., Féret, B., Klopfenstein, M., Grandgirard, E., Alunni, V., Cerciat, M., Jung, M., Mayere, C., et al. (2023). Loss of nr5a1 in sertoli cells after sex determination changes their cellular identity and induces their death by anoikis. *bioRxiv*, pages 2023–01.

Taddei, M., Giannoni, E., Fiaschi, T., and Chiarugi, P. (2012). Anoikis: an emerging hallmark in health and diseases. *The Journal of pathology*, 226(2):380–393.

Yang, L., Lin, M., Ruan, W.-j., Dong, L.-l., Chen, E.-g., Wu, X.-h., and Ying, K.-j. (2012). Nkx2-1: a novel tumor biomarker of lung cancer. *Journal of Zhejiang University SCIENCE B*, 13:855–866.

Yin, T., Chen, S., Wu, X., and Tian, W. (2017). Genepanda—a novel network-based gene prioritizing tool for complex diseases. *Scientific reports*, 7(1):43258.

Yoo, J., Kim, J., Yoon, H., Kim, G., Jang, C., and Kang, U. (2021). Accurate graph-based pu learning without class prior. In *2021 IEEE International Conference on Data Mining (ICDM)*, pages 827–836. IEEE.

Zhai, Z., Lin, Z., Meng, X., Zheng, X., Du, Y., Li, Z., Zhang, X., Liu, C., Zhou, L., Zhang, X., et al. (2024). Disignatlas: an atlas of human and mouse disease signatures based on bulk and single-cell transcriptomics. *Nucleic Acids Research*, 52(D1):D1236–D1245.
